# Diagnostic, progressive and prognostic performance of m^6^A methylation RNA regulators in lung adenocarcinoma

**DOI:** 10.7150/ijbs.39046

**Published:** 2020-03-25

**Authors:** Zhizhi Zhuang, Liping Chen, Yuting Mao, Qun Zheng, Huiying Li, Yueyue Huang, Zijing Hu, Yi Jin

**Affiliations:** 1 Department of Rheumatology and Immunology, The Second Affiliated Hospital and Yuying Children's Hospital of Wenzhou Medical University, Wenzhou 325000, Zhejiang Province, China.; 2 Department of Pharmacy, Sir Run Run Shaw Hospital, School of Medicine, Zhejiang University, Hangzhou, Zhejiang 310000, China.; 3 Second clinical college of medicine, Wenzhou Medical University, Wenzhou 325000, Zhejiang Province, China.; 4 Department of Respiratory medicine, The Second Affiliated Hospital and Yuying Children's Hospital of Wenzhou Medical University, Wenzhou 325000, Zhejiang Province, China.; 5 Department of Hematology and Medical Oncology, The Second Affiliated Hospital and Yuying Children's Hospital of Wenzhou Medical University, Wenzhou 325000, Zhejiang Province, China.; 6 College of Pharmaceutical Sciences, Wenzhou Medical University, Wenzhou 325000, Zhejiang Province, China.

**Keywords:** N6-methyladenosine, diagnostic score model, risk score, clinicopathological characters, lung adenocarcinoma

## Abstract

**Background:** N6-methyladenosine (m^6^A) RNA methylation is dynamically and reversibly regulated by methyl-transferases ("writers"), binding proteins ("readers"), and demethylases ("erasers"). The m^6^A is restored to adenosine and thus to achieve demethylation modification. The abnormality of m^6^A epigenetic modification in cancer has been increasingly attended. However, we are rarely aware of its diagnostic, progressive and prognostic performance in lung adenocarcinoma (LUAD).

**Methods and Results:** The expression of 13 widely reported m^6^A RNA regulators in LUAD and normal samples were systematically analyzed. There were 12 m^6^A RNA methylation genes displaying aberrant expressions, and an 11-gene diagnostic score model was finally built (Diagnostic score =0.033*KIAA1429+0.116*HNRNPC+0.115*RBM15-0.067* METTL3-0.048*ZC3H13-0.221*WTAP+0.213*YTHDF1-0.132*YTHDC1-0.135* FTO+0.078*YTHDF2+0.014*ALKBH5). Receiver operating characteristic (ROC) analysis was performed to demonstrate superiority of the diagnostic score model (Area under the curve (AUC) was 0.996 of training cohort, P<0.0001; AUC was 0.971 of one validation cohort-GSE75037, P<0.0001; AUC was 0.878 of another validation cohort-GSE63459, P<0.0001). In both training and validation cohorts, YTHDC2 was associated with tumor stage (P<0.01), while HNRNPC was up expressed in progressed tumor (P<0.05). Besides, WTAP, RBM15, KIAA1429, YTHDF1, and YTHDF2 were all up expressed for TP53 mutation. Furthermore, using least absolute shrinkage and selection operator (lasso) regression analysis, a ten-gene risk score model was built. Risk score=0.169*ALKBH5-0.159*FTO+0.581*HNRNPC-0.348* YTHDF2-0.265*YTHDF1-0.123*YTHDC2+0.434*RBM15+0.143*KIAA1429-0.200*WTAP-0.310*METTL3. There existed correlation between the risk score and TNM stage (P<0.01), lymph node stage (P<0.05), gender (P<0.05), living status (P<0.001). Univariate and multivariate Cox regression analyses of relevant clinicopathological characters and the risk score revealed risk score was an independent risk factor of lung adenocarcinoma (HR: 2.181, 95%CI (1.594-2.984), P<0.001). Finally, a nomogram was built to facilitate clinicians to predict outcome.

**Conclusions:** m^6^A epigenetic modification took part in the progression, and provided auxiliary diagnosis and prognosis of LUAD.

## Introduction

According to Global Cancer Statistics 2018, there will have been approximately 18.1 million new cancer cases and 9.6 million death cases worldwide 1. Researchers around the world are constantly striving to improve the medical technology for providing more sensitive method for tumor diagnosis and effective approach for tumor therapy. However, due to the complexity of tumor formation mechanism, it is far from enough to understand the nature of cancer from the genetic layer in the traditional sense 2. It is recognized that the expression of oncogene depends not only on the gene itself, but also on epigenetic modification without changing gene sequence 2,3.

Epigenetics is a research hotspot in recent years, which is defined as no change in DNA sequence, but with heritable changes in gene expression 3. Previously, the epigenetic researchers are mainly focused on DNA and histone modifications. It was even believed that, for example, mRNA only played the role in information transmission. However, with the rapid development of high-throughput sequencing technology, it is found that mRNA also undergo various modifications such as N6-methyladenosine (m^6^A), N1-methyladenosine (m^1^A) and pseudouridine methylation during the process of exon splicing, 5'-capping and 3'-tailing 4-6. These modifications will then affect mRNA splicing, nucleation, stabilization, translation and other mRNA metabolism processes, thereby regulating gene expression. Up to now, 171 RNA modifications have been discovered 7. Researches on m^6^A epigenetic modification have been increasingly attended. M^6^A is a methylation modification that occurs on RNA adenine (A), and is one of the most abundant modifications in most eukaryotic mRNAs and long-chain non-coding RNAs (lncRNAs) 8-10. In transfer RNA (tRNA), ribosome RNA (rRNA), microRNA, the m^6^A methylation is likewise detected 11. Similar to DNA and protein modification, the m^6^A methylation is dynamically and reversibly regulated by methyl-transferases ("writers"), binding proteins ("readers"), and demethylases ("erasers") 12. The RNAs undergo the process of methyl group modification under the action of "writers" that such as METTL3, METTL14, WTAP, RBM15, KIAA1429, and ZC3H13 13-19. Then the "readers" including YTHDF1, YTHDF2, YTHDC1, YTHDC2, HNRNPC recognize those m^6^A-modified RNAs and function in RNA processing, nuclear export, translation and decay 12,17,20. Relying on the "Erasers" (FTO, ALKBH5), the m^6^A is restored to adenosine and thus to achieve demethylation modification 21. Once the component involved in the regulation of m^6^A modification has been lost, the physiological functions such as cell differentiation and embryo development would be affected and expression of genes would be abnormally regulated 4,22.

At present, some diseases, such as obesity, diabetes, neuronal disease, infertility, autoimmune diseases, have been reported associated with m^6^A modification 23-28. In addition, m^6^A methylation is likewise closely related to the tumor development. M^6^A-related protein is an important regulator of tumorigenesis, high or low expression level often directly determines the tumor pathological process. For example, in cervical squamous cell carcinoma (CSCC), FTO is significantly high expressed 29. It would lower the m^6^A methyl level and in turn activate the beta-catenin pathway and affect ERCC1 expression and then lead to poor prognosis. In colorectal cancer, YTHDF1 is highly expressed associated with the tumor diameter (P=0.009), lymph node metastasis (P=0.044), distant metastasis (P=0.036) and clinical stage (P=0.0226) 30. As for lung cancer, report has indicated that FTO enhances the expression of myeloid zinc finger 1 (MZF1) by reducing m^6^A levels, thereby promoting the development of lung squamous cell carcinoma (LUSC) 31. Another evidence points that miR-33a inhibits the proliferation of non-small cell lung cancer (NSCLC) cells by targeting METTL3 mRNA 3'UTR binding sites 32. Thus, the fully understanding of the pivotal m^6^A RNA methylation regulators is vital for lung cancer treatment. In all categories of lung cancers, lung adenocarcinoma (LUAD) is one of the most common histological types 33. However, it lacks a comprehensive analysis of the expression of m^6^A RNA methylation regulators in lung cancer, especially in LUAD. The clinicopathological characteristics, diagnosis and prognostic value of such regulators remains to be explored.

In this study, the expression of 13 widely reported m^6^A RNA regulators in LUAD and normal samples were systematically analyzed. The different clinicopathological characters which related to each m^6^A modification regulator were then provided. It was found that the expression of m^6^A RNA methylation regulators played a responsible role in the progression of LUAD, which even were identified as the effective diagnostic and prognostic factors.

## Methods

### Datasets

Genotype-Tissue Expression (GTEx) dataset for normal lung tissues was downloaded from https://www.gtexportal.org/ 34. Data of lung adenocarcinoma and squamous cell lung carcinoma of TCGA 35 was downloaded from UCSC Xena (https://xena.ucsc.edu/) 36. For GTEx and TCGA dataset, RNA-sequencing data (FPKM values) were normalized into log_2_ (FPKM+1). Clinical data of TCGA was downloaded from cBioPortal (http://www.cbioportal.org/) 37,38. Data of GSE75037 39, GSE63459 40, GSE29013 41, GSE30219 42, GSE37745 43, and GSE50081 44 were downloaded from the Gene Expression Omnibus (https://www.ncbi.nlm.nih.gov/geo/).

After normalization by "LiMMA-normalizeBetweenArrays" (http://www.bioconductor.org/), GSE75037 and GSE63459 were served as validation cohorts of diagnostic model. GSE29013, GSE30219, GSE37745, and GSE50081 were merged by Batch normalization to serve as validation cohorts of prognostic risk model. The details of expression data were shown in Table 1. GTEx and TCGA dataset were acted as training cohort, and GSE75037, GSE63459, GSE29013, GSE30219, GSE37745, and GSE50081 as validation cohort. For GTEx and TCGA dataset, we defined expression value as log_2_ (FPKM+1), while for gene chips, expression value was defined as log_2_(expression).

### Selection of investigative genes

There were 13 m^6^A RNA methylation genes brought into the study, namely, METTL3, METTL14, WTAP, KIAA1429, RBM15, ZC3H13, YTHDC1, YTHDC2, YTHDF1, YTHDF2, HNRNPC, FTO, and ALKBH5 45,46. We investigated their role in diagnosis, progression, and prognosis of lung adenocarcinoma of both training and validation cohort.

### Diagnostic role of 13 m^6^A RNA methylation genes in lung adenocarcinoma

After normalization by "LiMMA-normalizeBetweenArrays", 288 samples of normal lung tissues from GTEx were added into TCGA dataset of lung cancer to increase the number of the normal group. Then wilcox test was applied to differential analysis of 13 m^6^A RNA methylation genes between normal and tumorous samples. Package "corrplot" was used to analyze the correlation among each gene. App ClueGO (http://www.ici.upmc.fr/cluego/version2.3.3) of Cytoscape (http://cytoscape.org, version3.5.1) was to illuminate the relationship between m^6^A RNA methylation genes and related pathways 47-48. Then the least absolute shrinkage and selection operator (lasso) regression model was made to diagnose lung adenocarcinoma in the training cohort by the “lars” package 49-50. Eventually, 11 m^6^A RNA methylation related genes and coefficients were identified with smallest 10-fold cross validated mean square error in the training cohort.

Diagnostic score = 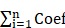
i*Xi 

(Coefi is the coefficient of each selected gene, Xi is the expression value.)

The diagnostic score model was further validated in the independent cohorts-GSE75037 and GSE63459. Besides, the distributions of diagnostic score were shown in different tumor stage in training and validation cohort to demonstrate the early-cancer discriminability of the diagnostic score model.

### Construction of the clusters stratified by m^6^A RNA methylation genes and correlation analysis

We used "ConsensusClusterPlus" to cluster tumor samples into two to five groups 51. Then Kaplan-Meier analysis and correlation analysis were applied to explain the correlation between clusters.

### Prognostic evaluation of 13 m^6^A RNA methylation genes in lung adenocarcinoma

To assess the prognostic evaluation of each m^6^A RNA methylation gene, univariate Cox regression analysis was performed. Besides, lasso regression model was also made with 13 m6A RNA methylation genes to optimize the prognostic meaning.

Risk score = 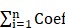
i*Xi

(Coefi is the coefficient of each selected gene, Xi is the expression value.)

From this risk score model, each patient could get a risk score. We defined patient into high risk group with the risk score≥ median value of all patients, and patient into low risk group with the risk score< median value. Then Kaplan-Meier analysis was performed to display the prognostic performance of Risk score model in both training and validation cohorts. And correlation analysis was applied to explain the correlation between subgroups stratified by the risk score model and clinicopathological characters, including smoking history, TNM stage, metastasis, lymph node stage, gender, diagnosed age, and status in both training and validation cohorts.

### Univariate and multivariate Cox regression analyses of clinicopathological characters and risk score

Univariate and multivariate Cox regression analyses of risk score and clinicopathological characters, including smoking history, TNM stage, metastasis, lymph node stage, gender, diagnosed age, and tumor stage, were performed to identify the prognostic performance of these characters. "Rms" package of R was used to build a nomogram with 1-year, 2-year, and 3-year overall survival (OS) as endpoints.

### Statistical analysis

All figures and data were accomplished by R (Version 3.5.3) and Graphad Prism 5.0 (La Jolla, CA). To estimate the diagnostic role of diagnostic model, operating characteristic (ROC) curves were plotted. One-way ANOVA or t-test was conducted to compare different clinicopathological characters in subclusters or risk groups. Kaplan-Meier plots, displayed as hazard ratio (HR) with 95% confidence intervals (CI), were performed to compare the OS of patients of different subclusters and risk groups. All results with P<0.05 were considered significant.

## Results

### Differential expression of m^6^A RNA methylation genes in lung adenocarcinoma

**Figure 1:**
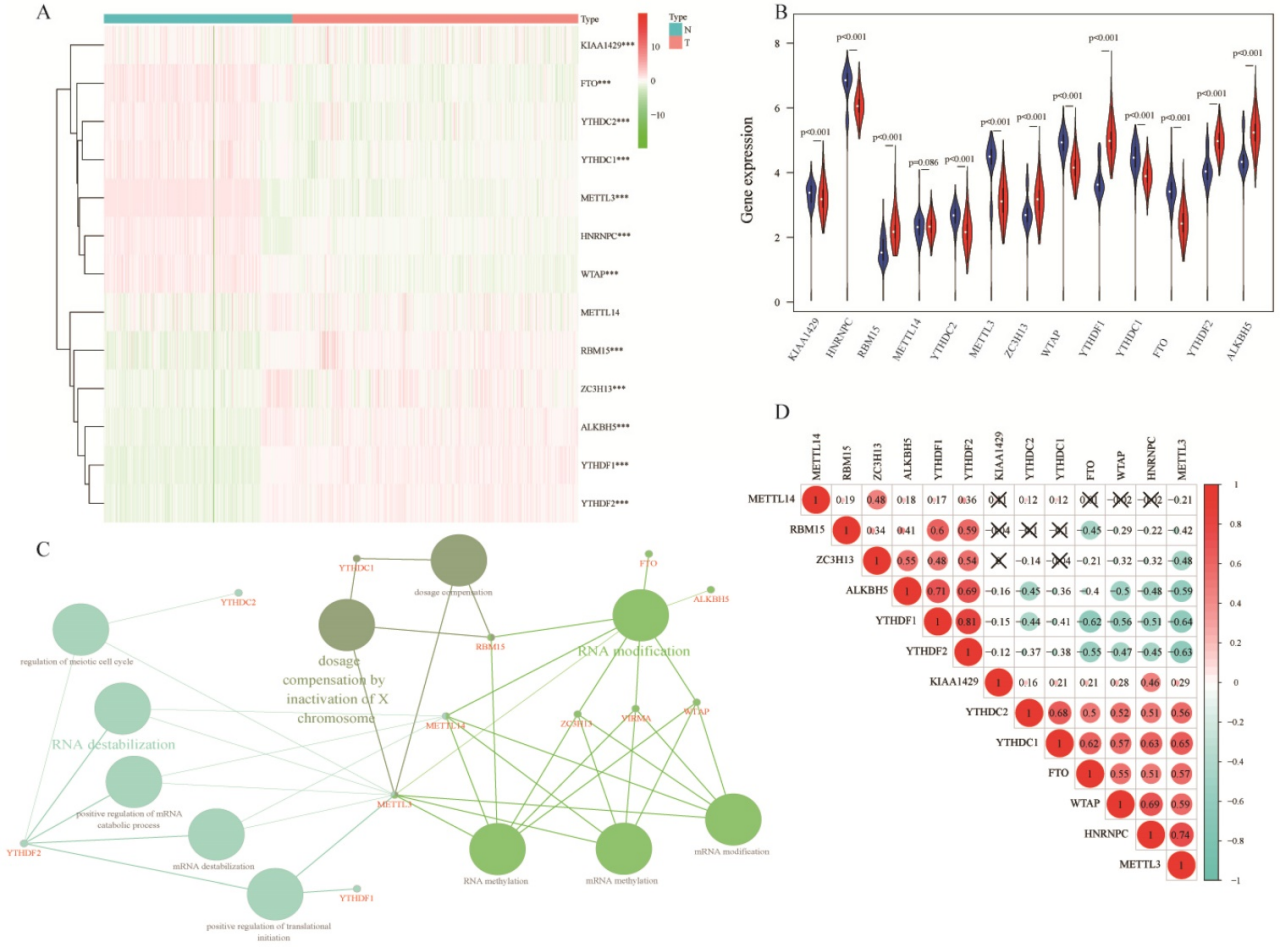
** Expression of m^6^A RNA methylation genes in lung adenocarcinoma.** (**A-B**) The differential expression of 13 m^6^A RNA methylation genes between 347 normal samples and 526 tumorous samples in lung adenocarcinoma. (**C**) Visualization of relation between m^6^A RNA methylation genes and related pathways. (**D**) Correlation analysis of 13 m^6^A RNA methylation genes. ***, P<0.001.

Comparing 13 m^6^A RNA methylation genes between 347 normal samples and 526 tumorous samples of TCGA and GTEx, we found that 12 of these genes had statistic difference. Among these genes, KIAA1429, HNRNPC, YTHDC2, METTL3, WTAP, YTHDC1, and FTO were down expressed in lung adenocarcinoma, RBM15, ZC3H13, YTHDF1, YTHDF2, and ALKBH5 were up expressed (Figure 1A-B). These 13 m^6^A RNA methylation genes were mainly associated with RNA modification, dosage compensation by inactivation of X chromosome, and RNA destabilization (Figure 1C). In spearman correlation analysis, YTHDC1, YTHDC2, FTO, WTAP, HNRNPC, and METTL3 were positively related with each other. Besides, ZC3H13, ALKBH5, YTHDF1, and YTHDF2 were also positively related with each other in lung adenocarcinoma. Furthermore, YTHDF1, and YTHDF2 were negatively related with FTO, WTAP, HNRNPC, and METTL3 (Figure 1D).

### Construction of diagnostic model

**Figure 2:**
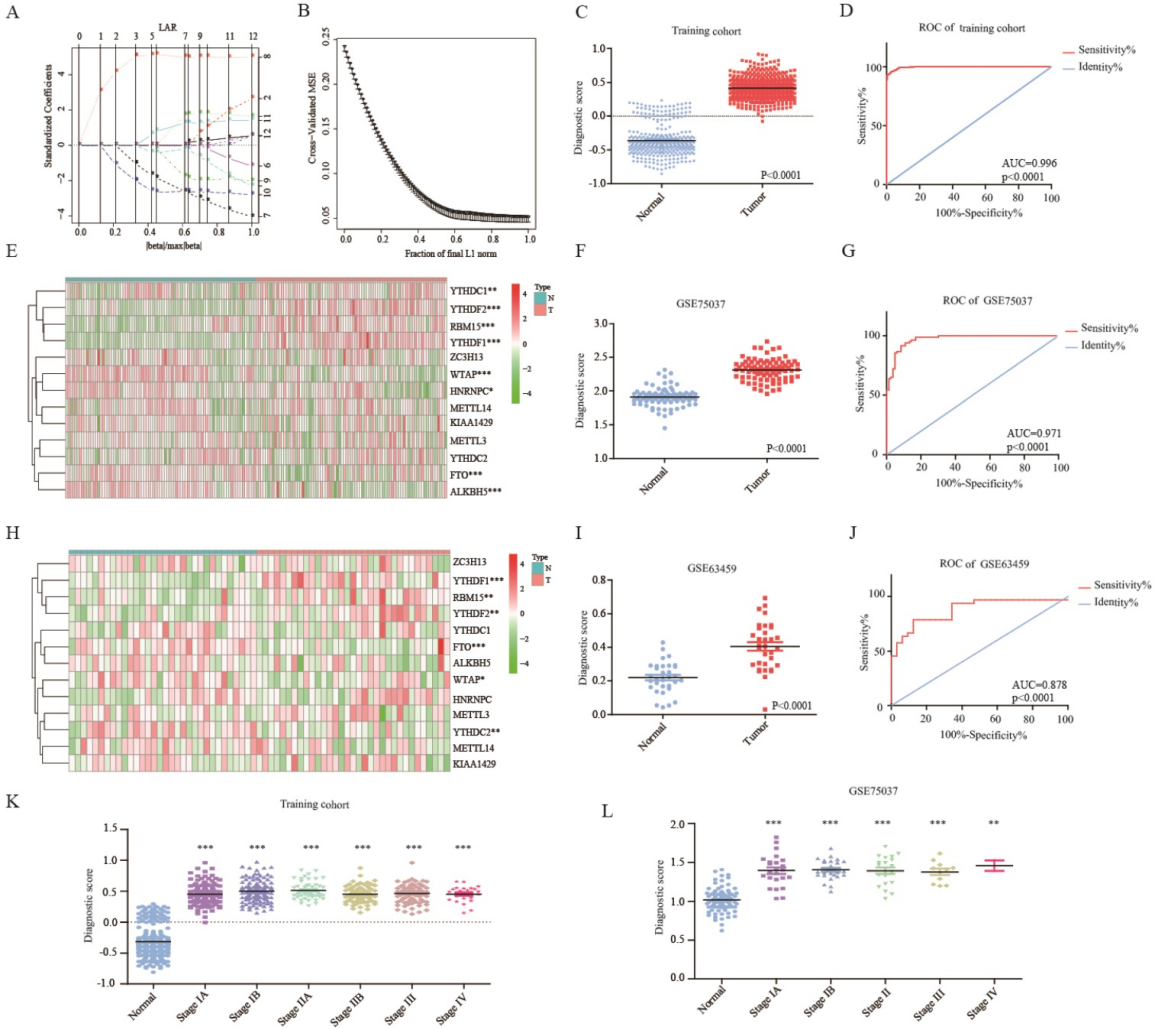
** Diagnostic role of m^6^A RNA methylation genes related model.** (**A-B**) The solution paths of lasso regression model and the relationship between cross-validated mean square error (CV MSE) and model size. (**C**) The difference in the distribution of diagnostic score of training cohort. (**D**) The ROC curve of diagnostic score of training cohort. (**E**) The expression of 13 m6A RNA methylation genes in lung adenocarcinoma of validation cohort-GSE75037. (**F-G**) The distribution and ROC curve of diagnostic score of validation cohort-GSE75037. (**H**) The expression of 13 m6A RNA methylation genes in lung adenocarcinoma of validation cohort-GSE63459. (**I-J**) The expression of 13 m6A RNA methylation genes in lung adenocarcinoma of validation cohort-GSE63459. (**K**) The difference in the distribution of diagnostic score of tumor stage of training cohort. (**L**) The difference in the distribution of diagnostic score of tumor stage of validation cohort-GSE75037. *, P<0.05; **, P<0.01; ***, P<0.001.

The ROC curves of 13 m^6^A RNA methylation genes were shown in Figure S1A. To improve the diagnostic accuracy, 13 genes were served as candidate genes into lasso regression analysis. Figure 2A-B showed the process of model building in the training cohort. Diagnostic score = 0.033*KIAA1429+0.116*HNRNPC+0.115*RBM15-0.067*METTL3-0.048*ZC3H13-0.221*WTAP+0.213*YTHDF1-0.132*YTHDC1-0.135*FTO+0.078*YTHDF2+0.014*ALKBH5. Each people could get a diagnostic score according to the diagnostic score model. The diagnostic scores of the tumor group were significantly higher than the normal group (Figure 2C). Area under the curve (AUC) was 0.996, P<0.0001, indicating high sensitivity and specificity (Figure 2D). The diagnostic score model was further validated in two independent cohorts-GSE75037 and GSE63459. Figure 2E and Figure S1B showed that the variation trends of HNRNPC, RBM15, YTHDF1, YTHDC1, FTO, and YTHDF2 of GSE75037 were accord with these in the training cohort. Meanwhile, people of tumor group also had extraordinarily higher diagnostic scores than these of normal group (Figure 2F). The AUC of diagnostic score model was 0.971, which was higher than single gene (Figure 2G and Figure S1C). For GSE63459, the trends of RBM15, WTAP, YTHDF1, FTO, and YTHDF2 were consistent with these in the training cohort (Figure 2H and Figure S1D). Likewise, the diagnostic score model had its superiority in diagnosing lung adenocarcinoma (Figure 2I-J and Figure S1E). Besides, diagnostic score model had discrimination of each stage of lung adenocarcinoma, even the very early stage-stage IA in the training and validation cohorts (Figure 2K-L).

### Correlation of m^6^A RNA methylation genes and clinicopathological characters

**Figure 3:**
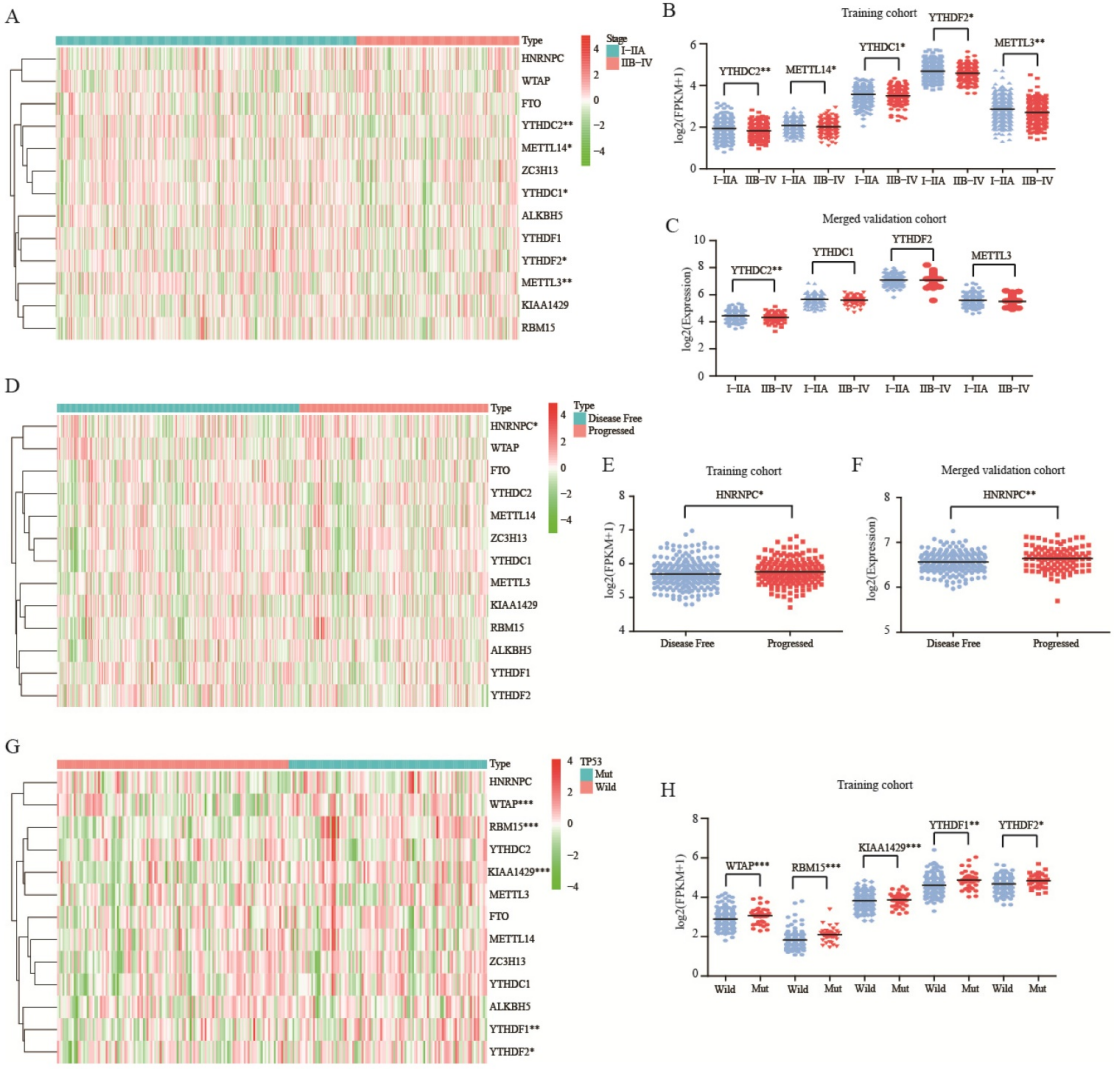
** Role of m^6^A RNA methylation genes in lung adenocarcinoma with different clinicopathological characters.** (**A-B**) The expression of m^6^A RNA methylation genes with different tumor stage of training cohort. (**C**) The expression of m^6^A RNA methylation genes with different tumor stage of merged validation cohort. (**D-E**) The expression of m^6^A RNA methylation genes with different tumor status of training cohort. (**F**) The expression of m^6^A RNA methylation genes with different tumor status of merged validation cohort. (G-H).The expression of m^6^A RNA methylation genes with TP53 mutation of training cohort.*, P<0.05; **, P<0.01; ***, P<0.001.

In both training and validation cohorts, YTHDC2 was associated with tumor stage (P<0.01, Figure 3A-C). Besides, HNRNPC was up expressed in progressed tumor group of training and merged validation cohorts (P<0.05, Figure 3D-F). For mutation, there was no correlation between m^6^A RNA methylation genes and KRAS or EGFR mutation (Figure S2). For TP53 mutation, WTAP, RBM15, KIAA1429, YTHDF1, and YTHDF2 were all up expressed in mutation group (Figure 3G-H).

### Construction of risk score model

**Figure 4:**
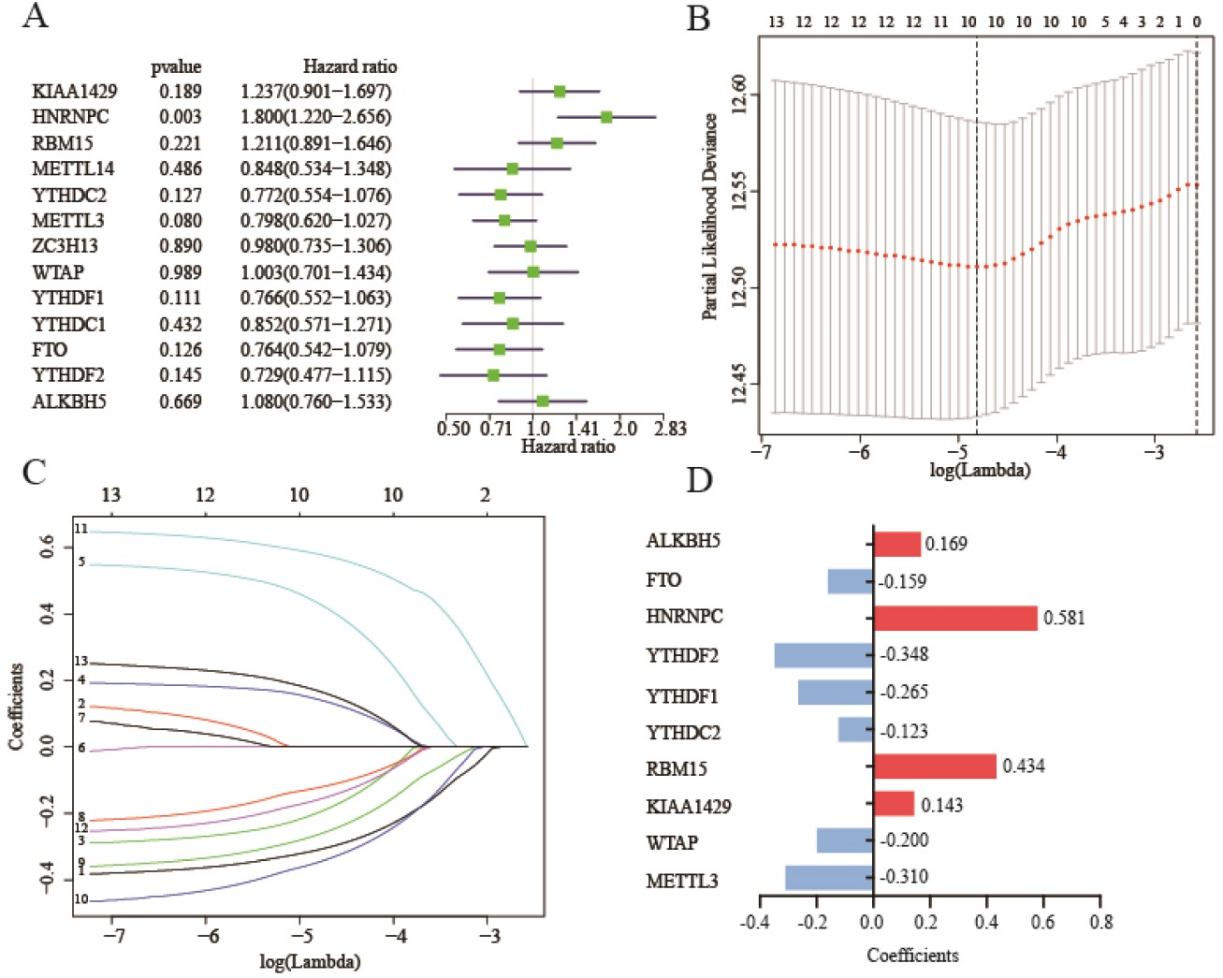
** Construction of risk score model with m^6^A RNA methylation genes.** (**A**) Univariate Cox regression analysis of 13 m^6^A RNA methylation genes. (**B-C**)The process of building lasso model with size and coefficients by multivariate Cox regression. (**D**) The coefficients of 10 m^6^A RNA methylation genes involve in lasso risk model. Hazard ratio (95% confidence intervals).

Before we build the risk score model, we clustered the patients into two to five subclusters (K=2-5) stratified by 13 m^6^A RNA methylation genes. Regretfully, subclusters stratified by m^6^A RNA methylation genes were not related with OS (Figure S3A-H). Due to subclusters that didn't have a good prognostic performance, we assessed the prognostic performance by a univariate Cox regression analysis in the training cohort. In these genes, HNRNPC was hazard of OS (HR: 1.8, 95%CI (1.22-2.656), P=0.003) (Figure 4A).

**Figure 5:**
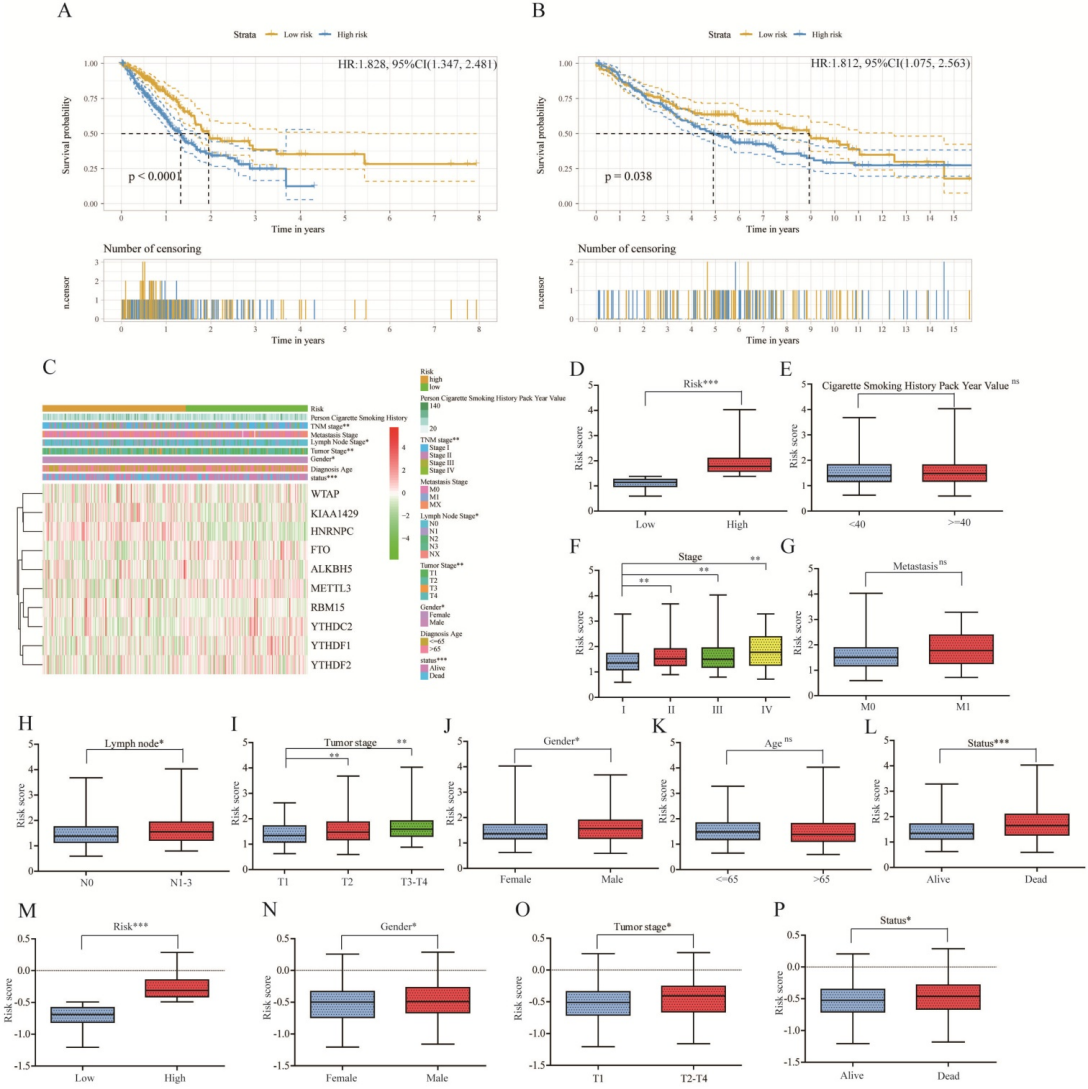
** Relationship between clinicopathological characters and risk score model in lung adenocarcinoma of training cohort and merged validation cohort.** (**A**) Kaplan-Meier plot of high risk and low risk subgroup in training cohort. (**B**) Kaplan-Meier plot of high risk and low risk subgroup in merged validation cohort. (**C**).The heatmap of 10 m^6^A RNA methylation genes involve in lasso risk model and relationship between clinicopathological characters and risk subgroup. (**D-L**) The distribution of risk score in different risk (D), smoking history (E), stage (F), metastasis (G), lymph node (H), tumor stage (I), gender (J), age (K), status (L) subgroup of training cohort. (**M-P**) The validation of distribution of risk score in different risk (M), gender (N), tumor stage (O), status (P) subgroup. *, P<0.05; **, P<0.01; ***, P<0.001, ns, no sense; HR, hazard ratio; 95%CI, 95% confidence intervals.

To better predict survival of lung adenocarcinoma with m^6^A RNA methylation genes, we conducted lasso regression analysis (Figure 4B-C). Then a ten-gene risk score model was built. Risk score=0.169*ALKBH5-0.159*FTO+0.581*HNRNPC-0.348*YTHDF2-0.265*YTHDF1-0.123*YTHDC2+0.434*RBM15+0.143*KIAA1429-0.200*WTAP-0.310*METTL3 (Figure 4D). We defined patient into high risk group with the risk score ≥ median value of all patients, and patient into low risk group with the risk score < median value. The training cohort (n=500) and merged validation cohort (n=350) were divided into high- or low-risk group respectively. Figure 5A-B showed that risk score model had its superior performance in predicting outcome of lung adenocarcinoma in both training (HR: 1.828, 95%CI (1.347, 2.481)) and validation cohort (HR: 1.812, 95%CI (1.075, 2.563)). Figure 5C showed the expression of 10 m^6^A RNA methylation genes involved in risk score model. There existed correlation between risk group and TNM stage (P<0.01), lymph node stage (P<0.05), gender (P<0.05), status (P<0.001). The distributions of risk score, smoking history, tumor stage, metastasis, lymph node stage, tumor stage, gender, diagnostic age, and status of training cohort were shown in Figure 5D-L. And the correlations between risk score and gender, tumor stage, status were verified in merged validation cohort (Figure 5M-P).

### Univariate and multivariate Cox regression analyses of clinicopathological characters and risk score

**Figure 6:**
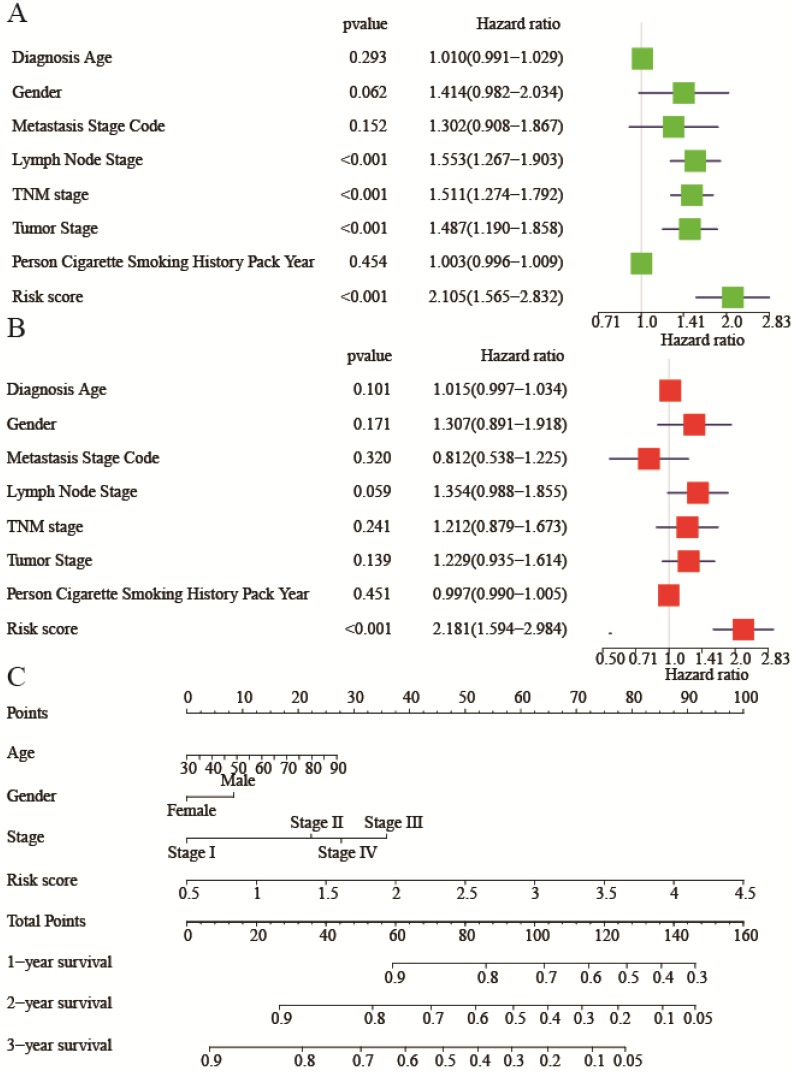
** Univariate and multivariate Cox regression analysis of clinicopathological characters and risk score.** (**A**) Univariate Cox regression analysis for evaluating the prognostic role of clinicopathological characters and risk score in lung adenocarcinoma. (**B**) Multivariate Cox regression analysis for evaluating the prognostic role of clinicopathological characters and risk score in lung adenocarcinoma. (**C**) The nomogram plot to predict 1-year, 2-year, 3-year overall survival. Summing up each point of clinicopathological characters and risk score to predict overall survival. Hazard ratio (95% confidence intervals).

To investigate whether risk score was an independent risk factor, we implemented univariate and multivariate Cox regression analyses of relevant clinicopathological characters and risk score. In the univariate Cox regression analysis, lymph node stage, TNM stage, tumor stage, and risk score were all associated with bad OS (Figure 6A). While in the multivariate Cox regression analysis, only the risk score was related with bad OS (HR: 2.181, 95%CI (1.594-2.984), P<0.001) (Figure 6B), indicating risk score was an independent risk factor of lung adenocarcinoma. A nomogram was built to facilitate clinicians to predict outcome. Diagnostic age, gender, TNM stage, and risk score were given points according to their impacts to the outcome (Figure 6C). By summing up all the points, we could get a total point for each patient. Then we could be aware of 1-year, 2-year, and 3-year survival of patients respectively.

### Role of the diagnostic score model and prognostic risk model in squamous cell lung carcinoma

We investigated the expression of 13 m^6^A RNA methylation genes in squamous cell lung carcinoma. From Figure S4A-B, the variation trends of 12 m^6^A RNA methylation genes were in line with these in lung adenocarcinoma. Besides, the result of correlation analysis of squamous cell lung carcinoma was roughly consistent with that in lung adenocarcinoma (Figure S4C). The diagnostic score could diagnose squamous cell lung carcinoma as well (AUC=0.992, P<0.0001) (Figure S4D). However, the risk score that enabled clinicians to predict outcome of lung adenocarcinoma couldn't be applied to squamous cell lung carcinoma (Figure S4E).

## Discussion

Lung cancer is the most common malignant tumor with high morbidity and mortality worldwide, of which NSCLC accounts for 85-90%, including LUSC, LUAD and large cell lung cancer (LCLC) 52. The underlying cause of the low lung cancer survival rate is its difficulty in early diagnosis. More than 70% patients are diagnosed at an advanced and usually fatal stage, lacking the effective therapeutic measures 53. Thus, it becomes great significance to understand the development mechanism for its well diagnosis and treatment. Among these subtypes, LUAD is responsible for approximately 40% of all NSCLC cases 33. The early diagnosis and treatment can greatly improve the survival rate of patients with LUAD. Recently, emerging evidence has indicated that the development of lung cancer is both affected by genetic variation and epigenetic variation 54. Epigenetic regulates gene expression from multiple levels, including DNA methylation, RNA regulation, histone modification, and chromosome remodeling 55. However, up to now, in lung cancers, only FTO and METTL3 have been reported as potential targets for its diagnosis and treatment 31,32. In this study, except FTO and METTL3, we also found that KIAA1429, HNRNPC, YTHDC2, WTAP, YTHDC1, RBM15, ZC3H13, YTHDF1, YTHDF2, and ALKBH5 had differential expression in LUAD.

In exploring the clinical value of these differential expression regulators, it was found that the joint detection of these regulators was more significant for the diagnosis of LUAD in comparison to the detection of mostly single regulator. Such combination provided higher predictive accuracy of 99.6% (AUC) for LUAD, while the most single regulator was less than 90%. The diagnostic score model we constructed involved 11genes. From the results of Figure S1, in the training and validation cohorts, YTHDF1 had a good diagnostic performance. So far, many studies in progress with regard toYTHDF1 in colorectal carcinoma and hepatocellular carcinoma, which was usually regarded as an independent prognostic factor 56-58. However, there has been no related reports on LUAD, and no has been conducted as a diagnostic factor. Though the diagnostic score model had a unique superiority in the diagnosis of LUAD, we could use YTHDF1 alone to diagnose disease in consideration of operability and simplification.

In the absence of diagnostic characteristics, YTHDC2 was then found to be associated with tumor stage. Accurate pathological staging is the basis for setting on ideal treatment. Increasing researchers are also devoted in the research of molecular staging using gene or protein expression. Previously, in colon cancer, it has been found a significantly positive correlation between YTHDC2 expression and the tumor stage 59. However, there is still lack of information for LUAD. The finding thus will provide new insight into our understanding of the function of YTHDC2 in LUAD. HNRNPC, except as a joint regulator in the diagnostic score model, it even exhibited differential expression in progressed tumor group and disease free group. Many studies have suggested that several types of solid tumor cells including breast cancer, gastric cancer, and esophageal squamous cell carcinoma acquired generation and development characteristics through HNRNPC disorder 60-62. In lung epithelial cells, the stability of urokinase receptor (uPAR) mRNA was regulated by HNRNPC 63. Increased uPAR expression as well as stabilization of uPAR mRNA would contribute to the pathogenesis of lung inflammation and neoplasia 63. In this study, we preliminary confirmed that HNRNPC played a critical role in LUAD progression, which was expected to provide a potential therapeutic target.

The consensus clustering stratified by 13 m^6^A RNA methylation genes was not associated with the most clinicopathologic characters and survival. The inconsistent aberrated trends of these m^6^A RNA methylation genes might account for the frustrated utilization of the stratification. Then, we performed univariate Cox regression analysis of each selected m^6^A RNA methylation genes. We found that HNRNPC also was hazard of OS (HR: 1.8, 95%CI (1.22-2.656), P=0.003). As we have demonstrated, for breast cancer cells, the repression of HNRNPC could inhibit cell proliferation and tumor growth 60. In gastric cancer, HNRNPC has been identified as a prognostic and therapeutic marker 61. However, to the best of our knowledge, there has been rarely reported that HNRNPC was involved in the prognosis of LUAD.

As reported, a number of monogenic prognostic markers that were found significantly associated with prognosis, such as p53, HER2, and ERBB3 64. However, due to the heterogeneity of NSCLC, the genes involved might vary greatly among those different individuals 64. Thus, it greatly prompted the researchers to construct gene expression profiles composed of multiple prognostic genes for using in the patients risk stratification. For instance, Kratz et al. ever have constructed a prognostic model using 14 genes which divided the patients with NSCLC into low-risk, intermediate-risk, and high-risk groups 65. The results showed that the 5-year survival rate among three groups was significantly different, and the model exhibited satisfactory predictability in prognosis than traditional clinical pathology staging in multiple validation cohorts. Similarly, in this study, A ten-gene risk score model (Risk score=0.169*ALKBH5-0.159*FTO+0.581*HNRNPC-0.348*YTHDF2-0.265*YTHDF1-0.123*YTHDC2+0.434*RBM15+0.143*KIAA1429-0.200*WTAP-0.310*METTL3)was built. It stratified the OS of patients with LUAD into high and low risk categories, which exhibited entirely differences in survival. Following the discovery of these prognostic genes, the risk group distinguished through risk score was found existed correlation with TNM stage, lymph node stage, gender, status, and tumor stage in both training and validation cohort. Besides, these clinicopathological features were associated with bad OS, and risk score was an independent risk factor of LUAD. At present, TNM stage is commonly performed to assess the prognosis of patients. Due to the limited risk factors included in the TNM staging system, it is not possible to conduct a precise prediction for NSCLC. Validating and combining more clinical pathology and molecular biology risk factors to establish an effective prognosis model becomes an important direction for future research in the development of multidisciplinary comprehensive treatment. In this study, based on characteristics of diagnostic age, gender, TNM stage, and risk score, a nomogram was built to facilitate clinicians to predict outcome. To some extent, it was expected to address the issue of prognostic heterogeneity caused by single factor or insufficient risk factors analysis.

In spite of these indelible contributions, as the study went on, it was found that the above regulators exhibited no significant differential expression in KRAS and EGFR mutant LUAD. In TP53 mutant LUAD, the regulators including WTAP, RBM15, KIAA1429, YTHDF1, and YTHDF2 all were highly expressed. Thus, we inferred that those regulators were likely only effective in some types of LUAD, while the specific reasons required following elucidation. Besides, it was note-worthy that YTHDF2 was merely reported to be involved with TP53 in peripheral T-cell lymphoma and gastric cancer yet 66,67. YTHDF1 was functionally interactional with TP53 in gastric cancer 67. To the best of our knowledge, it was not clear yet that TP53 mutant impacted the expression of WTAP, RBM15, and KIAA1429, in which more evidences were required to elucidate their mechanistic correlation.

In addition to LUAD, our work also indicated that m^6^A shared connections with LUSC, and the joint detection also provided higher predictive accuracy of 99.2% (AUC). Unfortunately, as we observed, these m^6^A RNA methylation regulators exhibited no prognostic value in LUSC. LUAD is more likely to occur in women and non-smokers 68. LUSC is more common in older men and is closely related to smoking 69. It is likely that different pathological features determine the different prognostic effects exhibited by the same regulators, which is worth investigating in future studies.

In this study, due to the limited sample size of normal samples, we normalized the lung tissues of GTEx and increased the credibility and accuracy of differential expression analysis. The robust analyses of correlation were performed among m^6^A RNA methylation regulators and diagnosis, pathological features or prognosis. The sample number of the study approximately reached 1900, which we thought to be enough to offset some confounding factors.

## Conclusion

In conclusion, our work systemically elucidated m^6^A's progressive role in LUAD, and provided insights into its diagnostic and prognostic function. However, m^6^A's functional details and its relationship with specific gene mutations and other types of NSCLC in controlling tumor progression merit further investigation.

## Supplementary Material

Supplementary figures and tables.

## Figures and Tables

**Table 1:** The basic information of series in the study

Series accession numbers	Platform used	No. of normal samples	No. of tumorous samples	AJCC stage	Gender	Mean age, [min, max]	Region	Survival Outcome
GSE75037	Illumina Human WG-6 v3.0 expression beadchip	83	83	I:50; II:20; III:11; IV:2	Female:118 Male:48	68,[39,90]	USA	NA
GSE63459	Illumina Human Ref-8 v3.0 expression beadchip	32	33	I:28; II:5	Female:34 Male:31	66,[47,88]	USA	NA
GSE29013	Affymetrix Human Genome U133 Plus 2.0 Array	0	30	I:16; II:6; III:8	Female:10 Male:20	64,[32,76]	USA	OS
GSE30219	Affymetrix Human Genome U133 Plus 2.0 Array	0	85	NA	Female:19 Male:66	61,[44,84]	France	OS
GSE37745	Affymetrix Human Genome U133 Plus 2.0 Array	0	106	I:70; II:19; III:13; IV:4	Female:60 Male:46	63,[40,83]	Sweden	OS
GSE50081	Affymetrix Human Genome U133 Plus 2.0 Array	0	129	I:93; II:36	Female:62 Male:67	69,[40,86]	Canada	OS
TCGA-LUAD	Illumina RNAseq	59	526	I:172 II:273 III:45 IV:18 NA:18	Female:274 Male:237 NA:15	65,[33,88]	NA	OS
TCGA-LUSC	Illumina RNAseq	49	501	I:114 II:295 III:71 IV:21	Female:131 Male:370	67,[39,90]	NA	OS
GTEX	NA	288	0	NA	NA	NA	NA	NA

## Abbreviations

m^6^A: N6-methyladenosine
LUAD: lung adenocarcinoma
ROC: receiver operating characteristic
AUC: area under the curve
lasso: least absolute shrinkage and selection operator
m^1^A: N1-methyladenosine
lncRNAs: long-chain non-coding RNAs
tRNA: transfer RNA
rRNA: ribosome RNA
CSCC: cervical squamous cell carcinoma
MZF1: myeloid zinc finger 1
LUSC: lung squamous cell carcinoma
NSCLC: non-small cell lung cancer
GTEx: Genotype-Tissue Expression
OS: overall survival
HR: hazard ratio
CI: confidence intervals
LCLC: large cell lung cancer
uPAR: urokinase receptor
