# Supplementary material for: Diagnostic, progressive and prognostic performance of m6A methylation RNA regulators in lung adenocarcinoma
Source: Int J Biol Sci. 2020 Mar 25;16(11):1785–97. doi: 10.7150/ijbs.39046 (PMC7211177; doi:10.7150/ijbs.39046)
Supplement: Supplementary file 1 — Supplementary figures and tables. [file ijbsv16p1785s1.pdf]

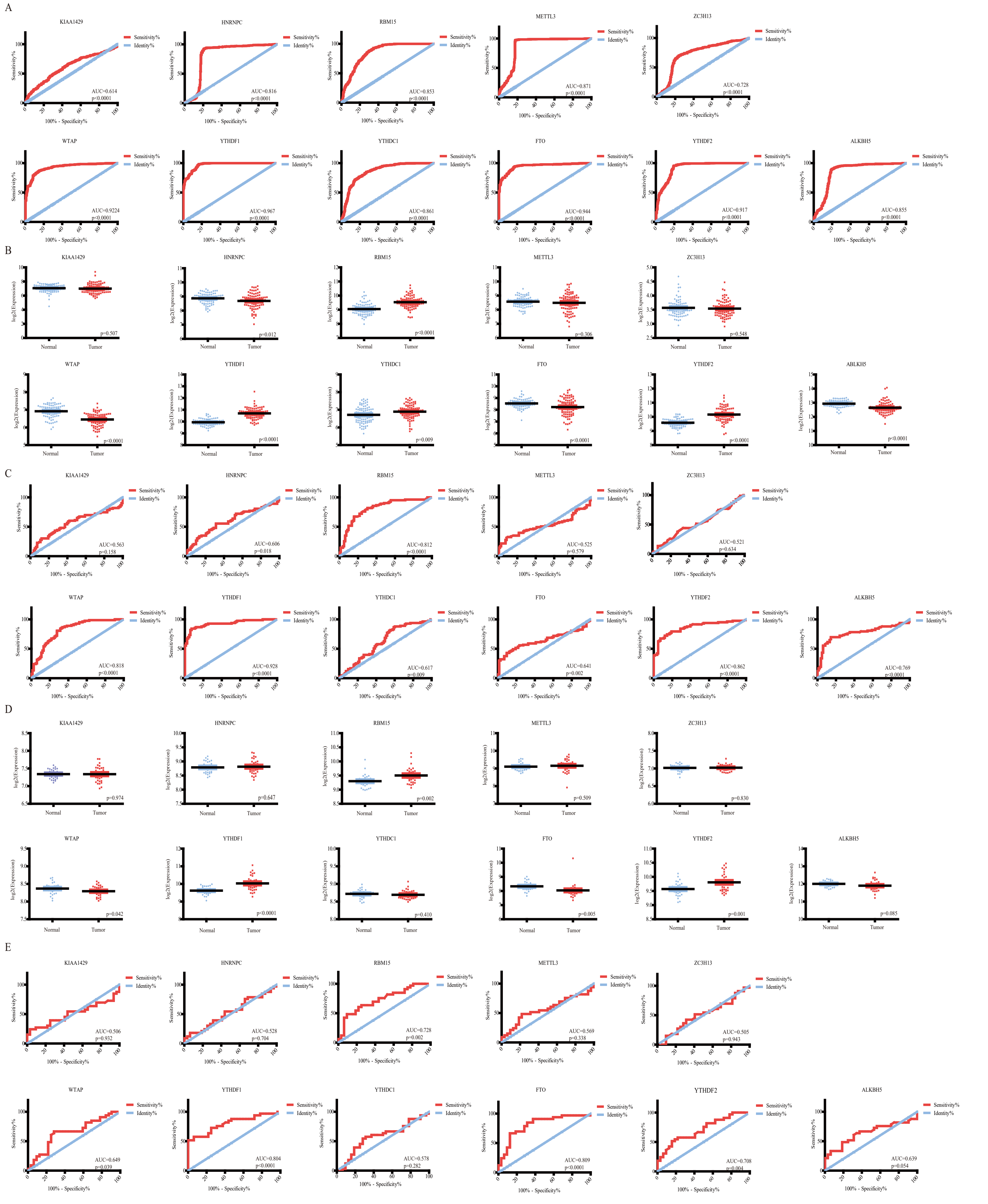

Figure S1. The ROC curve and distribution of 13 m6A RNA methylation genes of training and validation cohort. (A). The ROC curve of m6A RNA methylation genes of training cohort. (B-C). The distribution and ROC curve of m6A RNA methylation genes of validation cohort- GSE75037. (D-E). The distribution and ROC curve of m6A RNA methylation genes of validation cohort- GSE63459.

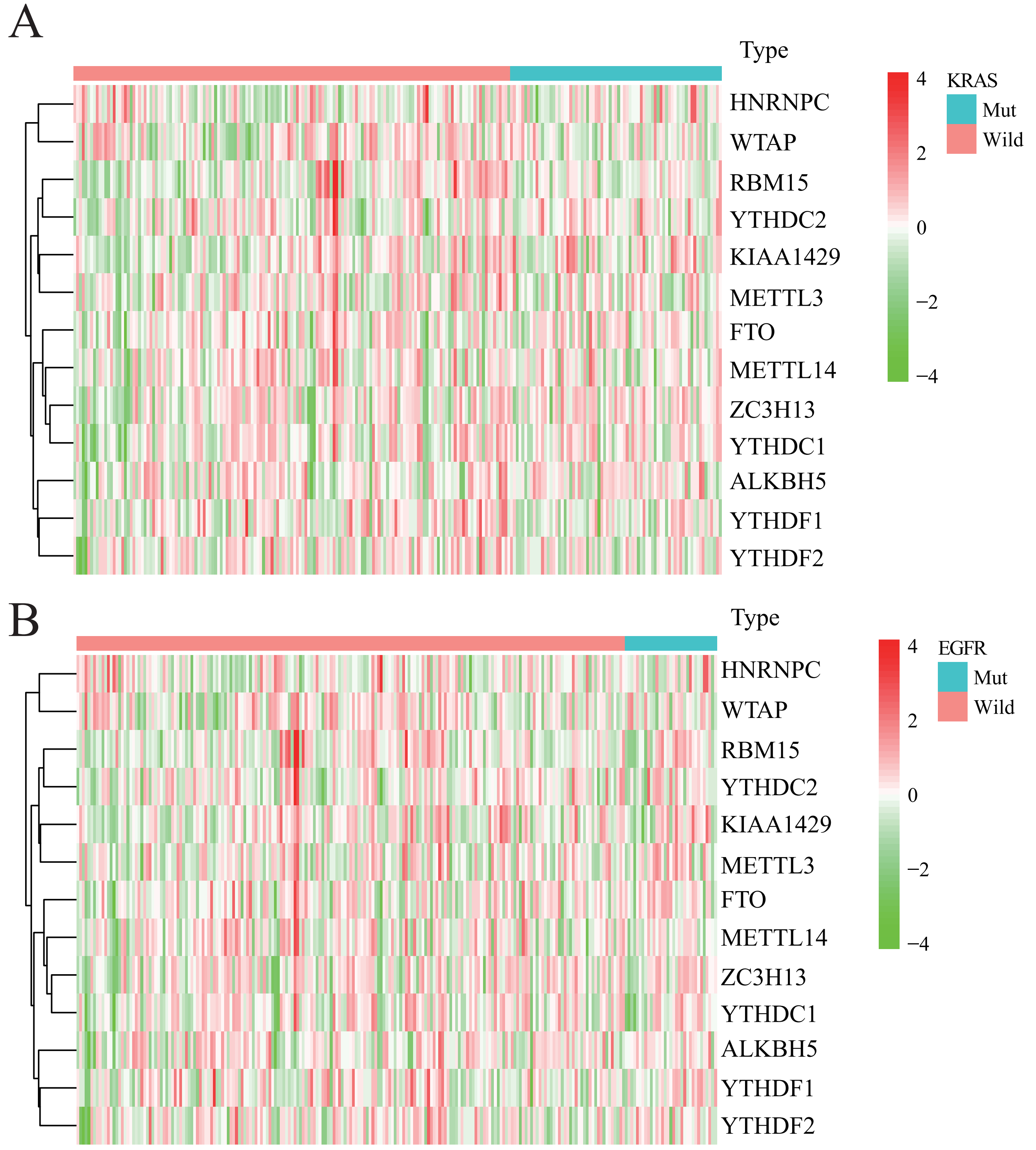

Figure S2. Role of m6A RNA methylation genes in lung adenocarcinoma with KRAS (A) and EGFR mutation (B).

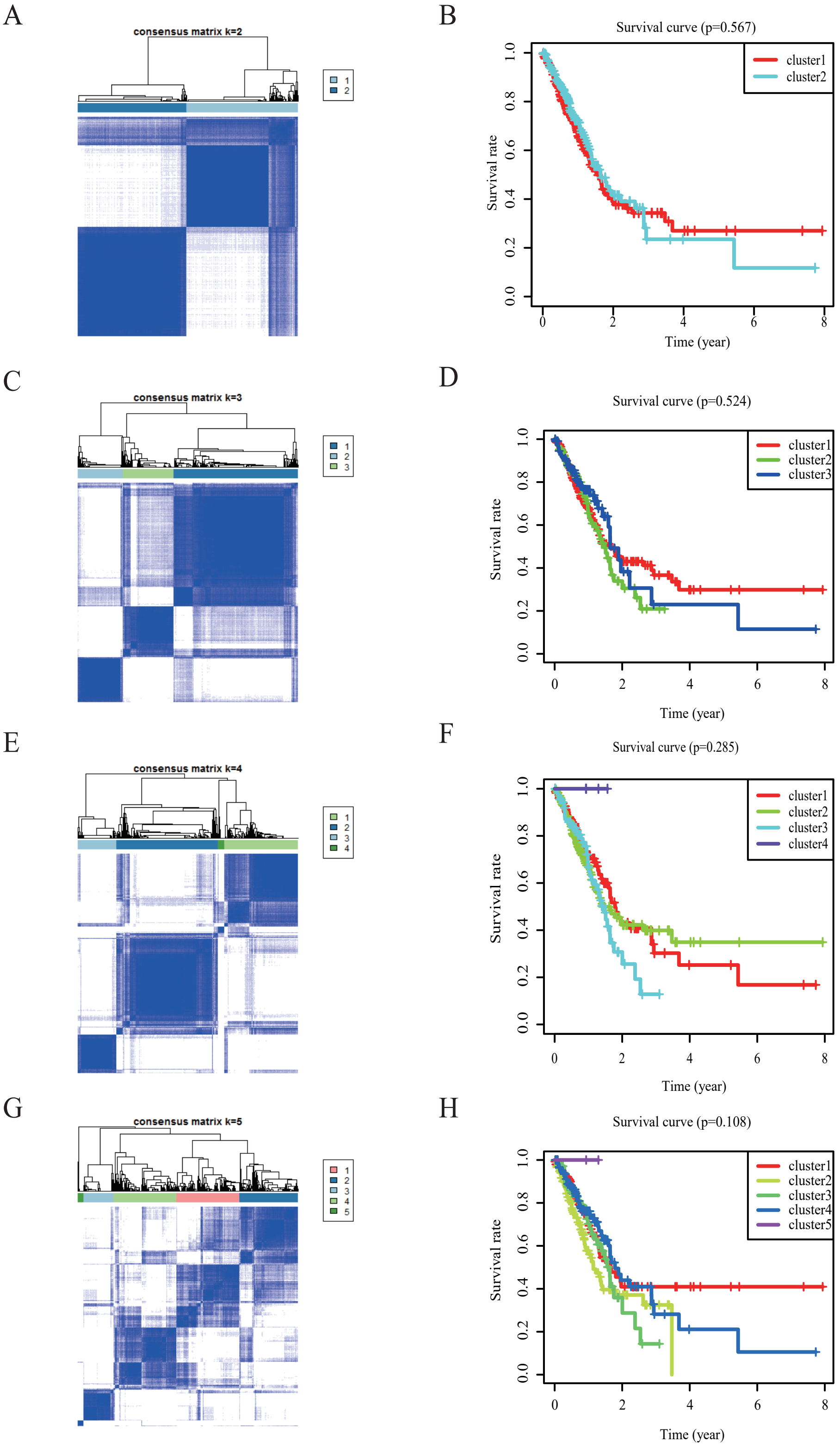

Figure S3. Construction of consensus matrixes of training cohort for each k (k=2-5) stratified by m6A RNA methylation genes and outcomes.

(A). Construction of consensus clusters stratified by m6A RNA methylation genes (K=2). (B). Kaplan-Meier plot of two clusters. (C). Construction of consensus clusters stratified by m6A RNA methylation genes (K=3). (D).Kaplan-Meier plot of three clusters. (E). Construction of consensus clusters stratified by m6A RNA methylation genes (K=4). (F). Kaplan-Meier plot of four clusters. (G). Construction of consensus clusters stratified by m6A RNA methylation genes (K=5). (H). Kaplan-Meier plot of five clusters.

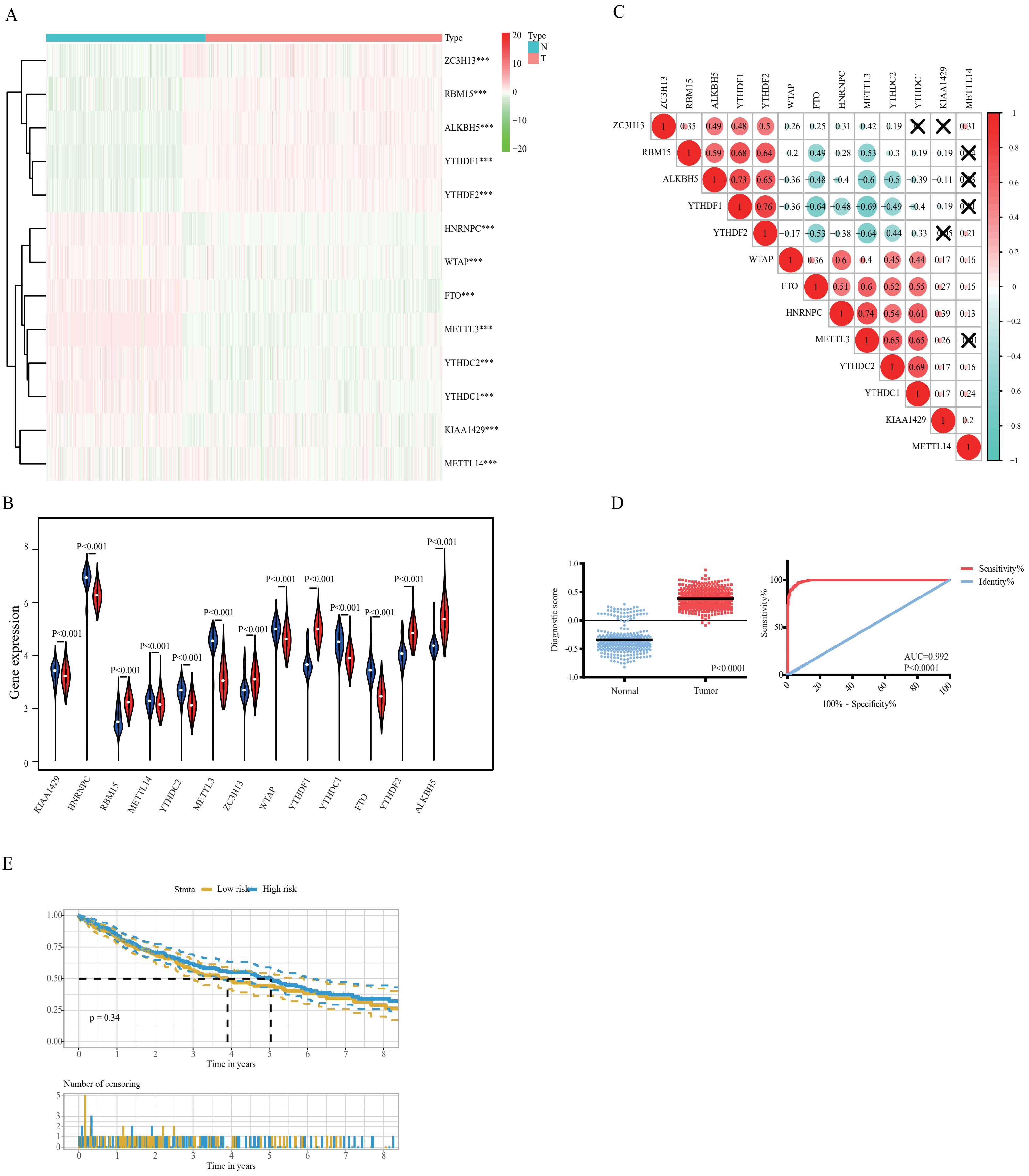

Figure S4. Expression of m6A RNA methylation genes and prognostic role of risk score model in squamous cell lung carcinoma.

(A-B). The differential expression of 13 m6A RNA methylation genes between 337 normal samples and 501 tumorous samples in squamous cell lung carcinoma. (C). Correlation analysis of 13 m6A RNA methylation genes. (D). Kaplan-Meier plot of the risk score model in squamous cell lung carcinoma. *, P<0.05; **, P<0.01; ***, P<0.001.
